# Compliance and persistence with daily, weekly, and monthly bisphosphonates for osteoporosis in Japan: analysis of data from the CISA

**DOI:** 10.1007/s11657-015-0231-6

**Published:** 2015-08-22

**Authors:** Hideaki Kishimoto, Masayuki Maehara

**Affiliations:** Department of Orthopedic Surgery, Nojima Hospital, 2714-1 Sesaki-machi, Kurayoshi, Tottori 682-0863 Japan; Ajinomoto Pharmaceuticals Co., Ltd., Tokyo, Japan

**Keywords:** Adherence, Bisphosphonate, Compliance, Osteoporosis, Persistence, Monthly dosing

## Abstract

**Summary:**

Compliance and persistence with daily, weekly, and monthly bisphosphonates (BPs) for osteoporosis were assessed using data from the Platform for Clinical Information Statistical Analysis (CISA) database that contains data of prescriptions in 13 university hospitals in Japan. The analysis revealed compliance and persistence improved as the dosing interval increases.

**Purpose:**

BPs are an effective first-line therapy for osteoporosis, but adherence is low. Compliance (medication possession ratio, MPR) and persistence (time to discontinuation) with daily, weekly, and monthly BPs were compared to ensure better adherence.

**Methods:**

Using data from the CISA database containing prescription data in 13 university hospitals in Japan, adherence to oral BPs of osteoporotic patients was investigated. Daily and weekly BPs were compared for compliance and persistence over 5 and 8 years, and daily, weekly, and monthly BPs for those over 1 and 2 years.

**Results:**

MPR over 5 years was 20.8 and 60.9 % for daily and weekly BPs (*p* < 0.001), respectively. MPR over 1 year was 38.6, 70.6, and 77.7 % for daily, weekly, and monthly BPs (*P* < 0.001), respectively. Persistence over 8 years was significantly higher in weekly than daily BPs (*p* < 0.001), and that over 5 years was highest in patients receiving BPs monthly (*p* < 0.01).

**Conclusion:**

The present analysis indicates that a monthly regimen has better adherence to treatment as compared with weekly and daily regimens.

## Introduction

Bisphosphonates (BPs) are an effective first-line therapy for the treatment of osteoporosis, but adherence to therapy could be lower than other drugs for the treatment of chronic diseases with the exception of gout treatment and others [1, 2]. The poor adherence to BP therapy has been related to the complexity of required dosing procedures and the severity of possible adverse drug reactions. As poor adherence to BP therapy has been reported to increase the risk of osteoporotic fractures [3, 4], better adherence must be ensured to continue treatment.

As a wide variety of BP products including those for daily, weekly, and monthly oral dosing, and those for monthly intravenous injection/infusion have been launched in Japan, physicians are able to select from diverse options to ensure better adherence to BP therapy. Although reports in Japan and other countries [5–7] have suggested better adherence with monthly than weekly dosing, there have been no published analyses using prescription databases in Japan.

Adherence to treatment is generally expressed with compliance, which is calculated as medication possession ratio (MPR), and persistence, which is defined as time to discontinuation [8]. In the present study, we used data from the Platform for Clinical Information Statistical Analysis (CISA) database that contains data of prescriptions in 13 university hospitals in Japan (http://www.cisa.jp) to calculate compliance (MPR) and persistence with oral BPs with different dosing intervals in osteoporotic patients.

## Patients and methods

Data from the CISA database were used to investigate adherence with oral BP therapy in patients with osteoporosis. Drug dispensing data at the individual level were retrieved from the CISA database, which contains substantial clinical information obtained in Japan. Data from CISA are provided in a fully anonymized form to 13 national university hospitals in this study. In the CISA database, each prescription record contains basic patient characteristics (anonymous identifier, gender, and date of birth) and information on the drug name, anatomical therapeutic chemical code, dosage, and dispensing date. Osteoporosis was diagnosed by Japanese guidelines which included the data of BMD, fracture history, and other risk factors [9].

Adherence to BP therapy was assessed with compliance and persistence. Compliance was measured with MPR, which was calculated by dividing the duration of prescription by the duration of observation. Persistence was calculated as the ratio of patients receiving BP therapy to patients prescribed with BPs at each time point. According to a previous report [6], a limit on the number of days allowed between refills, the permissible gap, was specified as 30, 30, and 45 days for daily, weekly, and monthly BPs, respectively [6] and 90 days for all in additional analysis [10].

### Compliance

Data of 12,230 patients who were newly prescribed with oral BPs during the period between April 2006 and August 2008 were assessed for MPR over 5 years (Table 1). The patients consisted of 4178 and 8052 patients receiving BPs daily and weekly, and 2353 men and 9877 women. The mean age was 59.8 years.

**Table 1:** Baseline characteristics of subjects

	Compliance (MPR)		Persistence	
Observation period	5 years	1 year	8 years	2 years
Date of first prescription	Apr 2006–Aug 2008	Nov 2011–Jan 2013	Apr 2006–Jan 2014	Nov 2011–Jan 2014
Daily	4178	242	6768	396
Weekly	8052	2516	15,204	4392
Monthly	–	2281	–	4538

Oral BPs for daily, weekly, and monthly dosing were launched in August 2001, September 2006, and September 2011, respectively

*MPR* medication possession ratio

Since oral BPs for monthly dosing were launched in September 2011, and became available in the 13 university hospitals in November 2011, a comparison of daily, weekly, and monthly BPs was made using data of 5039 patients who were newly prescribed with BPs during the period between November 2011 and January 2013 by calculating MPR over 1 year of daily, weekly, and monthly BPs. These patients consist of 242, 2516, and 2281 patients receiving BPs daily, weekly, and monthly, and 1150 men and 3889 women. The mean age was 62.0 years.

### Persistence

Persistence over 8 years was calculated using data from 21,972 ambulatory patients who were newly prescribed with oral BPs during the period from April 2006 to January 2014 (Table 1). These patients consisted of 6768 and 15,204 patients receiving BPs daily and weekly, and 4751 men and 17,221 women. The mean age was 60.8 years.

A comparison of persistence over 2 years with daily, weekly, and monthly BPs was made using data from 9326 ambulatory patients who were newly prescribed with oral BPs during the period from November 2011 to January 2014. Patients consisted of 396, 4392, and 4538 patients receiving BPs daily, weekly, and monthly, and 2066 men and 7260 women. The mean age was 62.2 years.

### Statistical analysis

Analyses in the present study were conducted in the CISA data center. The significance of the difference in MPR between two groups was tested using the Wilcoxon test, a non-parametric test, and that among three groups was tested using the Kruskal-Wallis test followed by the Bonferroni test to compare all pairs of the three groups. The persistent curve was estimated using the Kaplan-Meier method. The log-rank test was used to compare persistence between groups. All analyses were conducted using the statistical package R, version 3.1.2.

## Results

### Compliance

In 12,230 ambulatory patients who were newly prescribed with daily or weekly oral BP therapy during the period from April 2006 to August 2008, MPR over 5 years was 20.8 and 60.9 % for patients receiving BPs daily and weekly, respectively (*p* < 0.001) (Table 2).

**Table 2:** Mean MPR (%) for daily, weekly, and monthly BPs

MPR	Daily	Weekly	Monthly	*p* value
5 years	20.8	60.9	–	<0.001
1 year	38.6	70.6	77.7	<0.001

*MPR* medication possession ratio

MPR over 1 year in 5039 patients newly prescribed with daily, weekly, and monthly BP therapy from November 2011 to January 2013 was 38.6, 70.6, and 77.7 % in patients receiving the drug daily, weekly and monthly, respectively (*p* < 0.001). MPR was highest in patients receiving BPs monthly (Table 2).

### Persistence

**Fig. 1:**
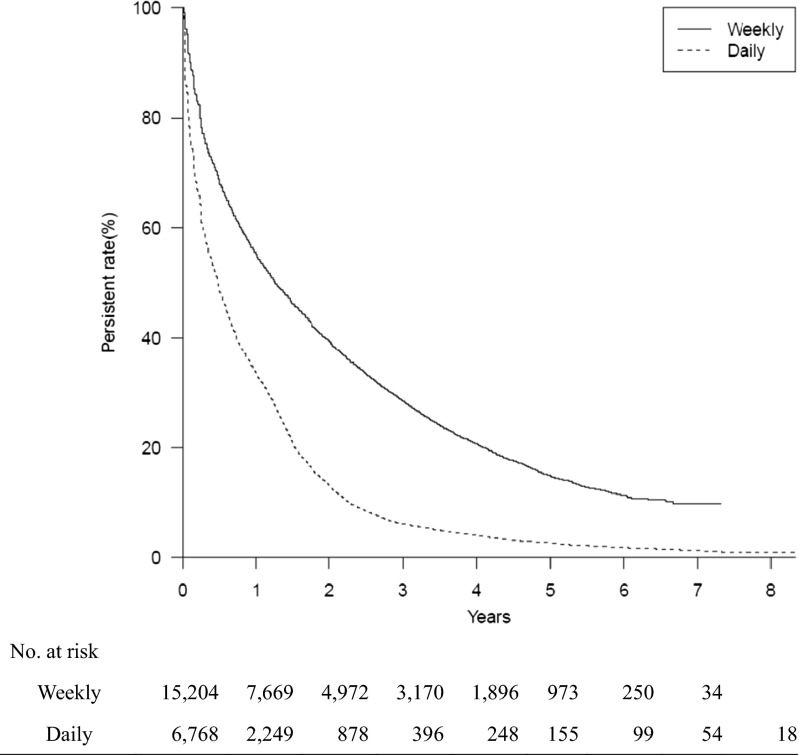
Kaplan-Meier analysis of the discontinuation of bisphosphonate treatment over 8 years (log-rank test; *p* < 0.001). A permissible gap of 30 days was allowed in this analysis

**Fig. 2:**
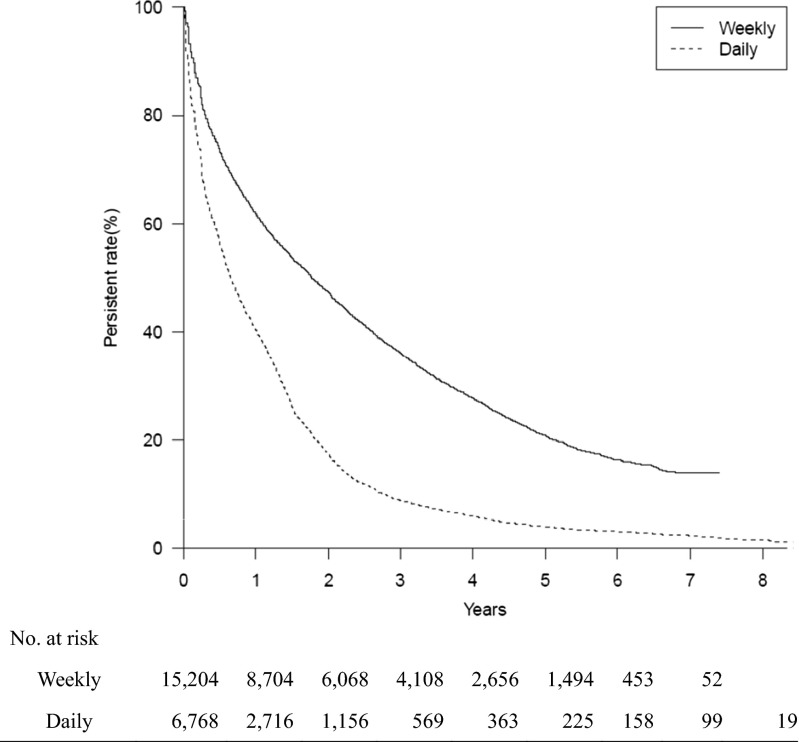
Kaplan-Meier analysis of the discontinuation of bisphosphonate treatment over 8 years (log-rank test; *p* < 0.001). A permissible gap of 90 days was allowed in this analysis

Figures 1 and 2 illustrate persistence over 8 years with daily and weekly BP therapy in 21,972 ambulatory patients who were newly prescribed with daily or weekly oral BP therapy during the period between April 2006 and January 2014. Persistence with permissible gaps of 30 and 90 days was significantly higher in patients receiving weekly than daily BP therapy at all time points (*p* < 0.001).

**Fig. 3:**
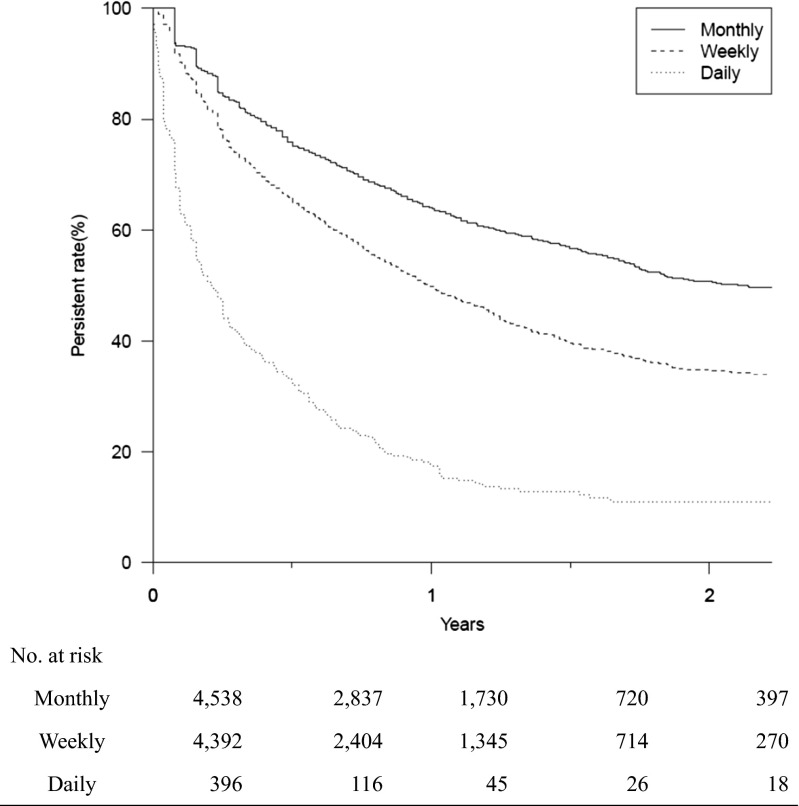
Kaplan-Meier analysis of the discontinuation of bisphosphonate treatment over 2 years (log-rank test, *p* < 0.001). A permissible gap of 30 days for daily/weekly and 45 days for monthly was allowed in this analysis

**Fig. 4:**
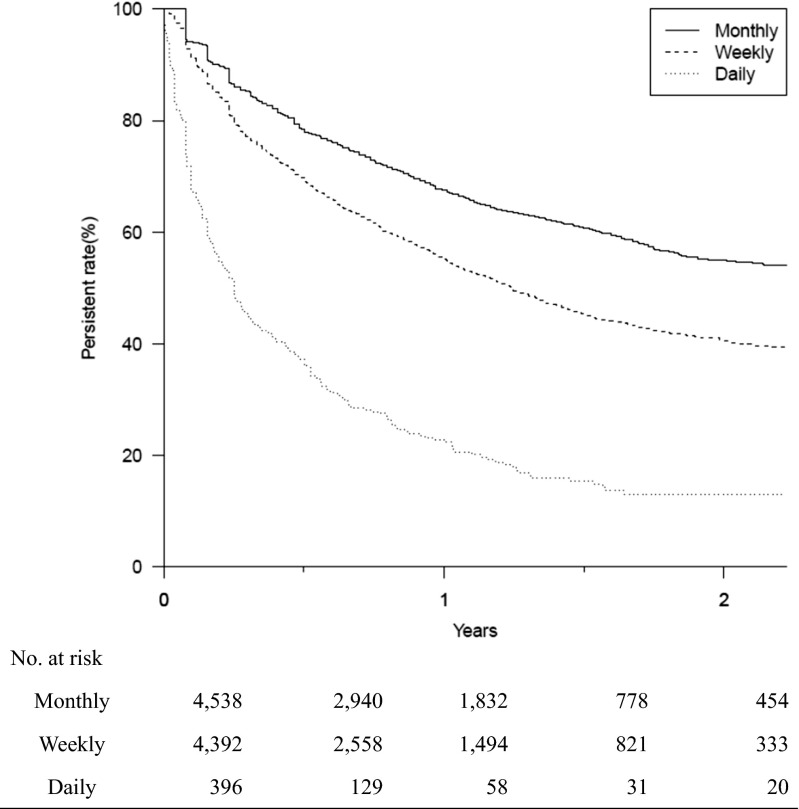
Kaplan-Meier analysis of the discontinuation of bisphosphonate treatment over 2 years (log-rank test, *p* < 0.001). A permissible gap of 90 days was allowed in this analysis

A comparison of persistence over 2 years with the three different dosing intervals in 9326 patients newly prescribed with oral BP therapy from November 2011 to January 2014 revealed that persistence was significantly higher in patients receiving BPs monthly throughout the 2-year period (*p* < 0.001) (Fig. 3). In an additional analysis using a permissible gap of 90 days, the persistence curve was shown with the same pattern as in Fig. 3 (Fig. 4).

## Discussion

Currently, a wide variety of BP products have been developed and are available in the clinical setting. Physicians are able to select from daily, weekly, or monthly oral therapy, or monthly intravenous injection/infusion to improve adherence to BP therapy. In the present study, we were able to calculate MPR over 5 years and persistence over 8 years to investigate the relationship between dosing interval of oral BPs and adherence to BP therapy in osteoporotic patients.

In previous studies where two dosing intervals of BPs were compared in Japan and other countries [5–7, 10–18], weekly and monthly regimens were superior to daily and weekly regimens, respectively, in terms of adherence. In the present study where daily, weekly, and monthly oral BP therapies were compared, adherence increased as the dosing interval increased.

Although the better adherence of monthly than weekly BPs is generally attributable to the convenience in using monthly BPs, it has also been reported that weekly BPs show persistence superior to monthly BPs [19] and that weekly and monthly BPs did not differ significantly in terms of persistence [20]. Researchers have pointed out that detailed explanation to the patient about the fracture prevention effects, possible adverse drug reactions, and safety of BP therapy is more important than dosing interval in terms of ensuring persistence [21–24]. Persistence of BP therapy should be improved not only through prolonging the dosing interval but through individualizing the treatment to meet the patient’s preference and circumstance and thereby motivate him/her to continue treatment [25–27].

As a difference in the permissible gap may result in different findings, an additional analysis using permissible gaps of 90, 90, and 90 days for daily, weekly, and monthly BPs was conducted in addition to the analysis with 30, 30, and 45 days according to a previous report [6]. Persistence was also higher for monthly BPs than daily and weekly BPs in the additional analysis.

It has been reported that MPR, a measure of compliance, of BP (alendronate or risedronate) closely correlates with the incidence of osteoporotic fracture, and that the incidence of fracture decreases substantially in patients with a MPR of >80 % although no fracture risk reduction was observed in patients with a MPR of <50 % [4]. As it has also been reported that for each decrease of the MPR by 1 %, the risk of hip fracture increased by 0.4 % [3], it is essential to encourage patients to continue treatment as instructed and maintain a high MPR, i.e., 80 % or higher, in order to prevent fractures. Compliance to treatment for osteoporosis is generally low [28]. In order to facilitate patients to understand the efficacy of treatment and continue treatment, drugs must exhibit noticeable effects such as improvement in bone mineral density [29]. In fact, BPs have favorable effects on bone mineral density that motivate patients to continue treatment. However, compliance with BP therapy must be improved further, although compliance increases as dosing intervals increase [11]. In the present study, MPR was highest in patients receiving BPs monthly, but MPR with monthly regimens over 1 year was 77.7 %, which did not exceed 80 %. Further approaches should be made to improve compliance [7, 28, 30].

One limitation of this study is that we could not obtain data on patient’s fracture history, details of examination results, and communication between healthcare professionals and patients in the clinical setting, such as explanation about the disease, from the CISA database we used to obtain prescription data. We could not assess the effects of communication between patients and healthcare professionals. Second, as the present study was conducted in patients who visited university hospitals for the treatment of osteoporosis, the results may not be generalized to patients treated in other settings. Thirteen national university hospitals are widely located without geographical deviation, but these data from CISA are not expressed for general Japanese data.

In conclusion, adherence with BP therapy was higher in monthly regimens than in daily and weekly regimens. Monthly regimens are considered beneficial in improving adherence to oral BPs for osteoporosis treatment.
